# Recognizing Belhassen Ventricular Tachycardia and Preventing Its Misinterpretation as Supraventricular Tachycardia: An Unusual Case Report

**DOI:** 10.7759/cureus.9817

**Published:** 2020-08-17

**Authors:** Anthony Furiato, Alexander Prestley, Abdul Waheed, Salvador Villanueva

**Affiliations:** 1 Emergency Medicine, Brandon Regional Hospital, Brandon, USA; 2 Surgery, Sandeman Provincial Hospital, Quetta, PAK

**Keywords:** belhassen ventricular tachycardia, bvt, verapamil-sensitive ventricular tachycardia, belhassen, verapamil-sensitive, anterior fascicle

## Abstract

Belhassen ventricular tachycardia (BVT), also known as verapamil-sensitive ventricular tachycardia, is an infrequent finding that can be fatal unless recognized early and treated in a prompt manner. Most patients have insignificant presentation suggestive of the disease, but on electrocardiography (EKG), BVT is characterized by a complete right branch block (RBB) and a right axis deviation (RAD). In this case report, we describe an unusual case of a 35-year-old male patient who presented to the emergency department (ED) complaining of acute palpitations of two-hour duration; subsequent diagnostic testing revealed BVT in the patient.

## Introduction

Belhassen ventricular tachycardia (BVT), or verapamil-sensitive ventricular tachycardia, is a subset of ventricular tachycardia that predominantly originates in the anterior fascicle [1]. First described in 1979, and then further studied by Dr. Bernard Belhassen and others in 1981, BVT is exquisitely sensitive to calcium channel blockers, specifically verapamil [1]. BVT mainly affects the male population in the age range of 15-40 years [2].

Although most of the patients presenting with BVT have some underlying structural anomalies in the heart, some patients have no underlying disease, which makes the diagnosis this pathology very challenging [2]. In most of the patients, the underlying etiology in BVT is attributed to a structural abnormality in the Purkinje fibers or myocardial tissue in the ventricle with a concomitant episode of reentry tachycardia [2]. Additionally, most of the patients with BVT are symptomatic, and those who are symptomatic most commonly present with dizziness, fatigue, and dyspnea [3]. Most commonly, BVT presents on electrocardiography (EKG) as a right-bundle-branch block (RBBB) with a right axis deviation (RAD) pattern and a relatively narrow QRS complex compared to other ventricular tachycardias [4]. It is important to note that the QRS complex is prolonged due to the origination of the electrical impulse but its morphology can make it appear much narrower on interpretation. Also, BVT is highly sensitive to verapamil. If it is administered promptly, BVT can be easily managed [5].

## Case presentation

**Figure 1:**
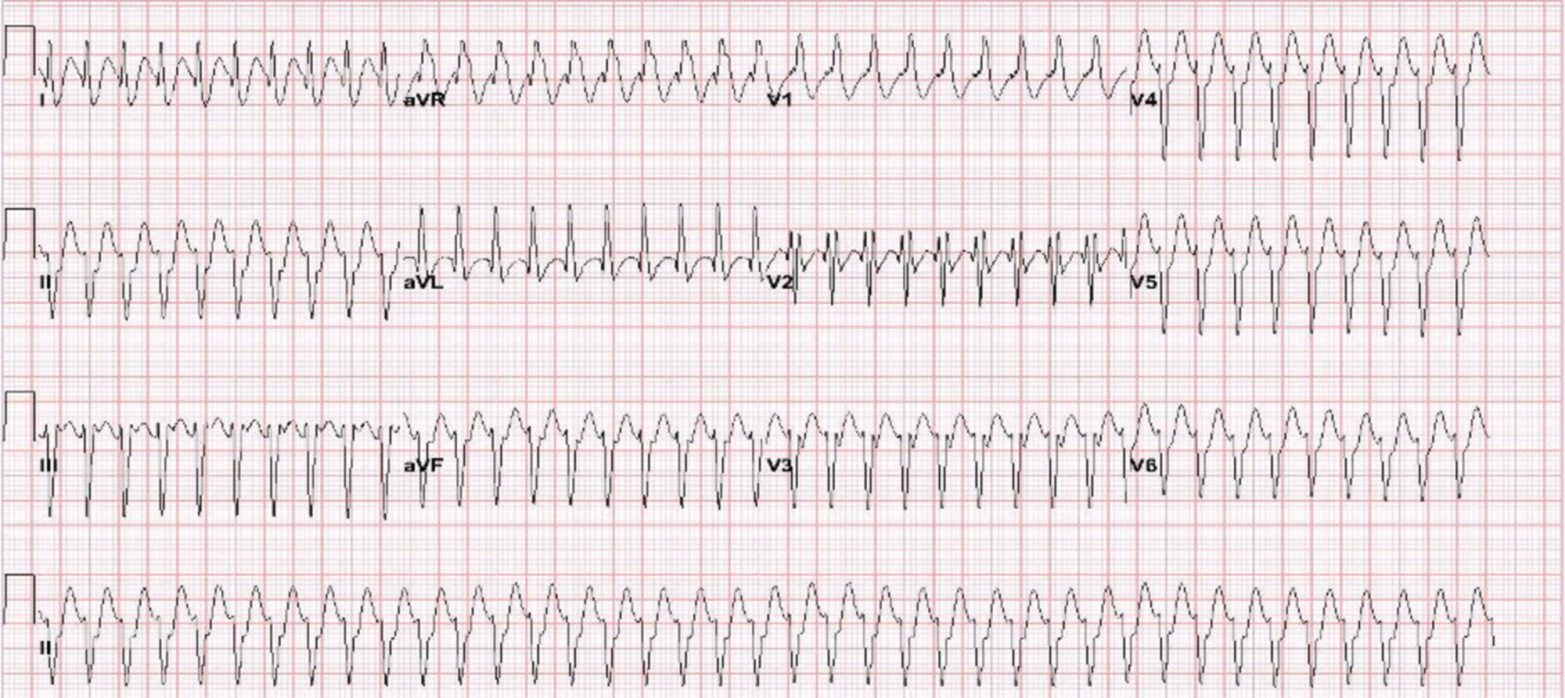
Electrocardiogram on arrival to the emergency department

A 35-year-old male with no significant past medical history presented to the emergency department (ED) with a chief complaint of palpitations of two-hour duration. EKG performed on arrival (Figure 1) revealed a regular wide-complex tachycardia suggestive of supraventricular tachycardia with bifascicular block. The patient’s vital signs on arrival were stable; therefore, a trial dose of adenosine was attempted, which had no effect on the patient.

Subsequently, the patient underwent procedural sedation with propofol and synchronized cardioversion at 100 joules, followed by 200 joules, with no change in rhythm. Further inspection of the EKG showed morphology consistent with RBBB and left anterior fascicular block. These findings were suggestive of anterior fascicular ventricular tachycardia, or BVT.

Additionally, the patient was treated with 2.5-milligrams verapamil intravenously (IV), which converted the aberrant rhythm to normal sinus rhythm. Further cardiac evaluation with troponin-I and echocardiogram were within normal limits. The patient was admitted to the cardiac care unit (CCU) for further monitoring and had an unremarkable hospital course with no further episodes of any arrhythmias.

The patient refused intra-cardiac electrophysiological study and ablation, and so he was discharged home two days later on 120 milligrams verapamil by mouth daily. Six months later, the patient experienced recurrent symptoms and agreed to the ablation procedure, which was successful in terminating the arrhythmia.

## Discussion

BVT poses a diagnostic challenge in the routine ED setting due to its non-specific nature of presentation [6]. It is also known as idiopathic ventricular tachycardia or, more commonly, fascicular or intra-fascicular tachycardia [2,6]. On EKG, BVT is usually characterized by an RBBB pattern and left axis deviation (LAD) (“Belhassen pattern”), which usually signifies its source of origin [2,6].

The clinical presentation of BVT is also challenging and can be easily confused with the other tachyarrythmias. Most patients suffering from this disease tend to be young and healthy [6,7]. Episodes of BVT are usually precipitated by some physical factors, emotional factors, or underlying infectious etiologies [6,7]. The initial presentation is commonly characterized by palpitations and shortness of the breath as the presenting complaints. These kinds of presentations carry an excellent prognosis [2,7]. Rarely, patients can present with syncope, and the prognosis in such patients is poor due to refractory arrhythmia progression [8].

BVT is most commonly triggered at set time intervals and easily by the premature stimuli [8]. This raises the suspicion that an underlying reentry phenomenon could be the possible cause of this tachycardia [2,6,7]. The reentry mechanism can be either a slow response action potential (SRAP) or a fast response action potential (FRAP) [1,6,8]. Due to the fact that BVT responds predominately to the verapamil, which more strongly inhibits SRAP, it is believed that this reentry phenomenon is mostly due to SRAP [2,4]. Furthermore, the automaticity theory due to delayed action potentials can also explain the occurrence of this syndrome [1,3].

Diagnosing BVT is also challenging and the EKG findings can be non-specific as well. Commonly, BVT presents on EKG as a minimally widened QRS complex, with or without a similar QRS configuration of the sinus rhythm or an RBBB pattern [5]. Moreover, the limb leads show either normal axis or RAD. It is believed that the RBBB pattern with either LAD (common) or RAD (uncommon) is highly suggestive of BVT [5].

The treatment plan for BVT is divided into both short-term and long-term management. IV verapamil is considered the gold standard in the initial emergent management, followed by long-term catheter ablation (CA) [9]. CA is effective in curing almost 90% of the disease in the long term [10]. In patients who are not willing to undergo CA, oral verapamil has been less successful for the permanent cessation of the arrhythmia but is likely the best option for outpatient management [5]. The role of adenosine has not been well established in the acute management of BVT [11]. Finally, if diagnosed properly and in a timely manner, the disease carries an excellent prognosis while tachycardia-related cardiomyopathy is exceedingly rare.

## Conclusions

BVT is more commonly found in young males, and it mostly presents with dizziness, palpitations, and dyspnea. The disease can be easily mistaken for other tachyarrhythmias. Paying special attention to the EKG pattern is essential for the precise and timely diagnosis of BVT. IV verapamil offers the quickest and most readily available emergent management, while CA offers the most effective long-term management of the condition.
